# Targeted Axillary Dissection with ^125^I Seed Placement Before Neoadjuvant Chemotherapy in a Danish Multicenter Cohort

**DOI:** 10.1245/s10434-023-13432-4

**Published:** 2023-04-16

**Authors:** Frederikke Munck, Inge S. Andersen, Ilse Vejborg, Maria K. Gerlach, Charlotte Lanng, Niels T. Kroman, Tove H. F. Tvedskov

**Affiliations:** 1 Department of Breast Surgery, Herlev-Gentofte Hospital, Gentofte, Denmark; 2 Department of Breast Surgery, Viborg Regional Hospital, Viborg, Denmark; 3 Department of Breast Examinations and Capital Mammography Screening, Herlev-Gentofte Hospital, Gentofte, Denmark; 4 Department of Pathology, Herlev-Gentofte Hospital, Gentofte, Denmark

## Abstract

**Background:**

Targeted axillary dissection (TAD), with marking of the metastatic lymph node before neoadjuvant chemotherapy (NACT), is increasingly used for breast cancer axillary staging. In the case of axillary pathological complete response (ax-pCR), axillary lymph node clearance can be omitted. Several marking methods exist, most using re-marking before surgery. Feasibility, learning curve, and identification rate (IR) vary. Marking with ^125^I seed before NACT makes re-marking at surgery redundant, possibly increasing feasibility and IR. Here, TAD with ^125^I seed placed before NACT is evaluated in a Danish multicenter cohort.

**Methods:**

Patients staged with ^125^I TAD in Denmark between 1 January 2016 and 31 August 2021 were included. Patients were identified in radioactivity-emitting implant registries at the radiology departments and from the Danish Breast Cancer Group database. Data were extracted from patients’ medical records. Information on patient/tumor characteristics, ^125^I seed activity, marking period, TAD success, number of sentinel nodes (SNs), the histopathological status of excised nodes, and whether the marked lymph node (MLN) was an SN were registered.

**Results:**

142 patients were included. The IR of the MLN was 99.3%, and the IR of the SLNB was 91.5%. TAD success was 91.5%. Minor challenges in marking or removal of the MLN were noted in three patients. In 72.3% of the patients, the MLN was a sentinel node. Overall, 40.8% had axillary pCR.

**Conclusion:**

TAD with ^125^I seed marking before NACT is feasible without re-marking at surgery and with only minor surgical challenges. The IR is high. Staging with TAD spares 41% of breast cancer patients an axillary dissection.

With the increasing use of neoadjuvant chemotherapy (NACT) in breast cancer treatment, the need for exact axillary nodal status must be balanced against minimizing surgery-related morbidity. NACT has been shown to induce axillary pathological complete response (ax-pCR) in 31–63% of patients, depending on tumor receptor profile.^1–8^ Patients with ax-pCR are not expected to benefit from axillary lymph node dissection (ALND).

Sentinel lymph node biopsy (SLNB) has been considered for axillary staging in node-positive patients following NACT. Studies have found an overall identification rate (IR) of the sentinel node (SN) of 80–89%, a false negative rate (FNR) of 14–17%, and a negative predictive value (NPV) of 57–86%.^9,10^ Despite an FNR above 10% being considered generally unacceptable, SLNB is recommended in some centers provided three or more SNs are removed and/or a dual tracer is used,^11,12^ as these measures have been shown to lower the FNR.^4,8,9,13^

Caudle et al. demonstrated in a pilot study that marking a positive lymph node before NACT and excision of the marked lymph node (MLN) along with SLNB after NACT was feasible. They reported an FNR of 0% in 9 patients receiving ALND.^14^ Simultaneously, Donker et al. demonstrated an IR of the MLN of 97% and an FNR of 7% with ^125^I seeds placed before NACT; however, the NPV of the MLN was 83%.^15^ These efforts facilitated the development of the targeted axillary dissection (TAD) method, where the positive lymph node is marked under ultrasound guidance before NACT and excised and evaluated along with the SN after NACT. Several new methods for marking the positive lymph node before NACT have been introduced. Currently, the choice of marking method depends on center preference and local legislation governing temporary implants. Coils, magnetic markers, ink tattooing, wire-guided localization, reflector-based systems, radiofrequency identification tags, and radioactive seeds are used. IR varies between marking methods and is reported to range from 78 to 100%.^16–21^

Often, one marker is placed at diagnosis, and when NACT is completed, it is necessary to place a different, intra-operatively identifiable marker. Studies report difficulties in identifying the MLN at the re-marking procedure in preparation for surgery.^22–24^ A different approach is to mark the lymph node with titanium seeds containing ^125^I before NACT. The seeds emit gamma radiation detectable by a gamma probe at surgery, provided a sufficient radioactive source is chosen. Marking with ^125^I seed before NACT makes re-marking at surgery redundant. Hypothetically, this will improve the feasibility of the TAD procedure and increase the IR of the MLN after NACT.

This cohort study aimed to investigate the feasibility and the IR of TAD in Danish breast cancer patients when ^125^I seeds are placed before NACT and left in situ for retrieval of the MLN upon surgery.

## Materials and Methods

### Patient Inclusion

TAD with ^125^I marking before NACT was used in three Danish hospitals, Herlev Hospital, Rigshospitalet, and Viborg Regional Hospital, at the time of the study. We included all patients with verified lymph node metastases, receiving NACT, and planned for TAD with ^125^I seed marking between January 1, 2016, and August 31, 2021.

At Herlev Hospital and Rigshospitalet (Capital Region, Denmark), marking with ^125^I seeds in breast and lymph nodes are prospectively registered locally to ensure removal and proper handling of all embedded seeds. Patients eligible for inclusion were identified from the radioactivity-emitting implant registries for ^125^I seeds kept at the Department of Radiology, Rigshospitalet, and the Department of Radiology, Herlev Hospital. A few eligible patients from the Department of Radiology, Rigshospitalet, were identified by manually searching through all diagnosis-related group codes covering the placement of a marker in either breast or lymph node. Patients from Viborg Regional Hospital (Central Denmark Region, Denmark) were identified from data retrieved from the Danish Breast Cancer Group database and retrospectively registered. Patients were not included if there was no surgical attempt at TAD.

Exclusion criteria were a history of ipsilateral axillary surgery of any cause, less than four cycles of NACT, and a history of ipsilateral breast cancer.

### Radiology

At diagnosis, patients were examined by breast radiologists, who performed mammography and ultrasound of the breast and axilla. Any visually or palpably suspicious lymph nodes were biopsied with either fine needle aspiration cytology (FNAC) or core needle biopsy (CNB). In the case of more than one suspicious lymph node, the most accessible one was biopsied. One ^125^I seed was placed in the lymph node either at the biopsy or after histo-/cytopathological evaluation of the biopsy, relying on visual identification of the biopsied lymph node.

Histo-/cytopathological confirmation of lymph node metastasis was mandatory for inclusion. Patients had to have either “malignant cells” or “cells suspicious of malignancy” on FNAC or, in cases where CNB was performed, carcinoma originating from the breast. The ^125^I seeds (IsoAid, LLC, Port Richey, Florida) were produced in batches and, upon delivery to the radiology departments, had an activity of 4 MBq per seed. Leftover ^125^I seeds from a particular batch are usually discarded when the activity of each seed falls below 2 MBq. This ensures sufficient radioactivity for identification perioperatively when taking the half-life of ^125^I of 60 days into account.^25^ In this series, ^125^I seeds with activity below 2 MBq at the time of marking were included if intended for long-term marking. Marking was done in all cases before or during the first few cycles of NACT.

### Surgery

An attempt at TAD was mandatory for inclusion in the study. TAD was defined as the excision of the MLN and simultaneous SLNB according to the definition made by Caudle et al.^14^ The use of a dual tracer with ^99m^Tc and Patent Blue is recommended in the Danish guidelines for the identification of SN after NACT. ^99m^Tc was injected before surgery, and Patent Blue was injected at the beginning of surgery, either peritumorally or in the retroareolar area. SNs and MLNs were identified with a gamma probe. Radioactive and blue nodes were defined as SNs. SN numbers reported include MLN with signs of tracer.

### Data Collection

Data on patient age, BMI, treatment year, histological diagnosis and size of breast tumor, receptor status, and size of suspicious axillary lymph nodes were recorded. Duration of marking period, ^125^I seed activity at the time of marking, neoadjuvant regimen, whether surgical removal of the ^125^I MLN was successful, complications associated with ^125^I seed marking and MLN excision, number of SNs removed, and whether the ^125^I MLN was identified as an SN or not were recorded as well. Data on the histopathological status of the MLN and SN and whether ALND was performed were all registered. Surgical removal of the MLN was recorded as successful if the ^125^I seed enabled excision of the MLN, regardless of whether the seed was found in or adjacent to the MLN. Metastases were classified according to AJCC 8th ed.^26^ In case of missing information on breast tumor biopsy histological diagnosis, the histological diagnosis of the surgical specimen was recorded. Tumor type could not be determined in patients achieving breast pCR if diagnostic biopsy did not specify carcinoma type, and these patients were categorized as “Other.” Ax-pCR was defined as no residual disease in the removed LNs. Male and female patients were included alike, provided they met all inclusion and no exclusion criteria. Since ethnic information is not systematically recorded in Danish medical files, these data were not retrieved. Data was partly prospectively registered and partly retrospectively retrieved from patient medical files and stored in a REDCap database (REDCap, Vanderbilt University, Nashville, Tennessee, US).

### Outcomes and Statistical Analysis

TAD was defined as successful if both the SN and the ^125^I MLN were identified during surgery, and this was the primary outcome. Secondary outcomes were difficulties associated with the ^125^I marking or ^125^I TAD surgery and the proportion of patients who achieved ax-pCR. Differences in SN IR according to the treatment center and patient BMI were analyzed by χ^2^ test. All statistics were calculated with R statistical software (R Core Team, 2021, Vienna, Austria).^27^

The Danish Data Protection Agency (j.no. P-2019-811) and the Danish Patient Safety Authority (j.no. 31-1521-208) approved the study.

## Results

A total of 150 female patients were identified through local ^125^I seed registries, diagnosis-related group codes, and data extracted from the Danish Breast Cancer Group database. Eight of these patients were excluded, leaving 142 patients in the analysis. Reasons for exclusion were (1) less than four NACT cycles in four patients, (2) a history of ipsilateral mastectomy or invasive disease in three patients, and (3) one patient was excluded because of an unknown social security number, resulting in missing data.

Fifteen patients (10.6%) were treated at Viborg Hospital, 94 patients (66.2%) at Rigshospitalet, and 33 patients (23.2%) at Herlev Hospital. The median patient age was 51 years, with a range of 26–82 years, and 126 (88.7%) had invasive ductal carcinoma in the breast. The remaining consisted of 13 patients where histological diagnoses were not obtainable, one patient harboring invasive lobular carcinoma, and two patients diagnosed with ductal carcinoma in situ in the breast. These two patients were confirmed to have lymph node metastases before NACT. The receptor status of the primary tumor is reported in Table 1. The median time between the placement of the ^125^I seed and surgery was 146.5 days, with a range of 101–272 days. The median number of excised SNs was two. In 37 patients (26.1%), three or more SNs were found on SLNB. Patient, tumor, and treatment characteristics are shown in Table 1.

**Table 1:** Clinicopathological features of 142 Danish breast cancer patients treated with ^125^I TAD

Clinicopathological feature	No. (median)	% (range)
Age (years)	(51)	(26–82)
BMI (kg/m^2^)	(24.8)	(18.3–44.1)
Center		
Herlev Hospital	33	23.2
Rigshospitalet	94	66.2
Viborg Hospital	15	10.6
Treatment year		
2019	15	10.5
2020	70	49.3
2021	57	40.1
Clinical TN stage		
cTisN1	2	1.4
cT1bN1	3	2.1
cT1cN1	26	18.3
cT2N1	96	67.6
cT3N1	15	10.6
Breast tumor histology		
Invasive ductal carcinoma	126	88.7
Other	16	11.3
Lymph node biopsy procedure		
FNAC	123	86.6
CNB	19	13.4
Histopathological diagnosis of lymph nodes		
Carcinoma	12	8.5
Malignant cells	125	88.0
Cells suspicious of malignancy	5	3.5
Neoadjuvant regimen		
Anthracycline / taxane combination	138	97.2
Anthracycline only	3	2.1
Platinum-based regimen	1	7.0
Receptor subtype		
ER^+^/HER2/*neu*^*+*^	38	26.8
ER^+^/HER2/*neu*^*–*^	54	38.0
ER^–^/HER2/*neu*^*+*^	27	19.0
ER^–^/HER2/*neu*^–^	23	16.2
Lymph node marking		
Lymph node size on ultrasonography (mm)	(17.00)	(5–49)
I^125^ seed activity at marking (MBq)	(2.541)	(1.613–4.218)
Marking period (days)	(146.5)	(101–272)
TAD procedure		
Dual tracer used for SLNB	128	90.1
No. of sentinel nodes excised	(2)	(0–7)
Concordance between SN and MLN	94	72.3

*TAD* Targeted axillary dissection, *FNAC* Fine needle aspiration cytology, *CNB* Core needle biopsy, *ER* Estrogen receptor, HER2/*neu* Human epidermal growth factor receptor 2, *SLNB* Sentinel lymph node biopsy, *SN* Sentinel node, *MLN* Marked lymph node

The IR of the ^125^I seed MLN after NACT was 99.3%. The MLN was not found in one patient, and since no SN was found either, the patient had completion ALND. The ^125^I seed was found in fibrotic tissue, and the pathology report stated that it was unclear whether the placement of the ^125^I seed was subpar or the LN had regressed aggressively, leaving the ^125^I seed without evidence of surrounding lymphatic tissue. In three additional patients, minor difficulties associated with the marking procedure or excision of the MLN were reported. These consisted of one patient where a second ^125^I seed was placed due to displacement of the first and two patients where the ^125^I seed was found adjacent to the MLN.

In 11 patients, the SN was not detected. Adding the abovementioned patient with non-identification of both MLN and SN, 12 patients had failed SLNB. The IR of the SN was thereby 91.5%. No difference in IR of SN was found between treatment centers. Neither was BMI > 30 associated with non-identification of SN.

The TAD procedure, with identification of both MLN and SN, was successful in 130 patients out of 142 attempts (91.5%).

Overall, 58 patients, corresponding to 40.8%, had ax-pCR. Of these, one patient had no evidence of metastases and no treatment response in the MLN. Ax-pCR rates, according to the receptor profile, were 44.7% for ER^+^/Her2/*neu*^+^ tumors, 13.0% for ER^+^/Her2/*neu*^–^ tumors, 85.2% for ER^–^/Her2/*neu*^+^ tumors, and 47.8% for ER^–^/Her2/*neu*^–^ tumors.

Eighty-four patients (59.2%) did not achieve ax-pCR. Of these, 68 patients (81.0%) had macrometastases in the MLN or SN, nine patients (10.7%) had micrometastases, and six patients (7.1%) had isolated tumor cells in the MLN or SN. The last patient was the patient with no detection of either MLN or SN, who had macrometastases found in the ALND specimen.

All patients with residual disease found in the MLN or SN had ALND except one, who declined. Adding the one patient with no detection of either the MLN or the SN, a total of 83 ALNDs were performed. Within this group, 32 patients had no further metastases detected in the ALND specimen. In 27 patients, 1–3 metastatic lymph nodes were found; in the remaining 24 patients, more than three metastatic lymph nodes were found.

Six patients had an MLN without metastases yet harbored metastases in the SN. Another 18 patients had an SN without metastases, but metastases were found in the MLN. The ability of each procedure (SLNB, excision of MLN, and TAD) to detect residual disease is shown in Table 2.

**Table 2:** Sentinel node, marked lymph node, and targeted axillary dissection detection rate and residual disease detection rate in 142 Danish **b**reast cancer patients receiving NACT

Detection rates	SLNB	Excision of MLN	TAD
Identification rate	91.5% (130/142)	99.3% (141/142)	91.5% (130/142)
Residual disease detection rate ^a^	79.5% (66/83)	94.0% (78/83)	100% (83/83)

^a^of 83 patients receiving ALND due to metastases

*NACT* Neoadjuvant chemotherapy, *SLNB* Sentinel lymph node biopsy, *MLN* Marked lymph node, *TAD* Targeted axillary dissection

**Fig. 1:**
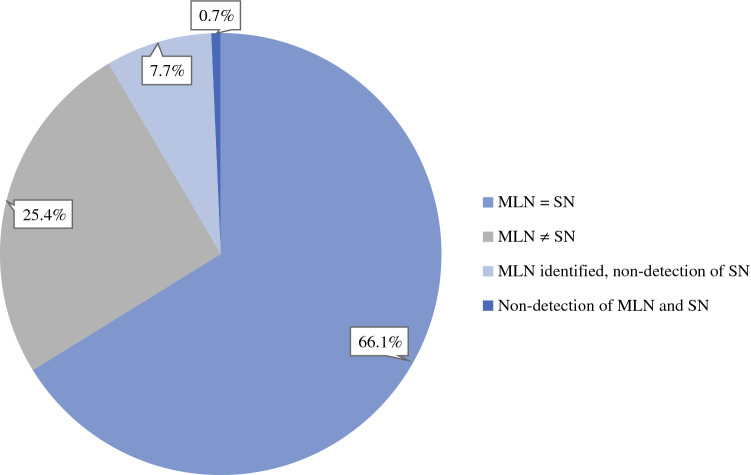
Marked lymph node (MLN) and sentinel node (SN) distribution and concordance in 142 node-positive Danish breast cancer patients treated with neoadjuvant chemotherapy (NACT) and staged by targeted axillary dissection (TAD) with one-step ^125^I seed marking

Of the 130 patients with successful SLNB, 94 had an MLN that displayed signs of tracer from the SLNB (Fig. 1). Thus, the concordance rate between MLN and SN was 72.3%.

## Discussion

We present here a very high IR of the MLN when performing TAD with ^125^I seeds placed before NACT. Of 142 patients, only one underwent ALND because no evidence of lymphatic tissue was found by the ^125^I seed, which raised concern that it might be a case of non-detection. This finding could, however, also represent a profound treatment response to NACT, resulting in identification in all cases. When choosing axillary staging with TAD, a high IR is crucial because non-detection of an MLN, which was known to be metastatic before NACT, leads to ALND to ensure proper staging and local control. This would impose a substantial risk of arm morbidity,^28,29^ despite knowing that ALND will be unnecessary in 41% with an ax-pCR in this study.

In addition to being a very reliable TAD marker, the ^125^I seed is easily managed in a surgical setting. In only three patients, minor difficulties were described in the patient file associated with the ^125^I seed marking procedure or surgical excision. Furthermore, ^125^I seed marking spares the patient an invasive radiological procedure before surgery known to carry discomfort, risk of complications, and interfere with the logistics surrounding surgery in the case of hook-wire placement.

Our results are in accordance with the IR of the MLN of 97% reported by Donker et al.^15^ Recently, the results from the RISAS trial were published. In this multicenter prospective cohort, including 227 patients, the IR of an SN was 86.4%, and the IR of an MLN was 94.1%. The FNR was 3.5%, and NPV was 92.8% for their combined TAD procedure (termed the “RISAS procedure”).^30^

Other marking methods exist that allow for the marker to be placed before NACT and retrieved upon surgery with success. One such is the reflector-based system SAVI SCOUT. In one study, this method had a reported IR of the MLN of 100%, but only 22 patients were included.^31^ A 100% IR with 0% FNR was also found by Martinez et al., who prospectively evaluated 44 patients with a Magseed® marker placed before NACT.^32^ However, the clinical significance of artifacts caused by implanted magnetic markers on NACT evaluation MRIs is undetermined. Marking with ^125^I seeds, on the other hand, causes no MRI artifacts that may hamper the assessment of axillary response on imaging, but are governed by local legislation and regulatory restrictions imposing varying difficulties with storage, handling, and disposal of the seeds.

A different one-step marking approach is presented in a recently published prospective trial where the suspicious lymph node is tattooed with a carbon suspension. This method relies on visual identification during surgery. The IR was reported to be 141 of 149 patients, i.e., 94.6%. However, in five patients, the identification was not made until ALND, rendering the IR closer to 91.3%. Furthermore, biopsy verification of nodal involvement before NACT was not obtained in all patients.^33^

The IR in one-step marking methods seems favorable compared with two-step marking procedures. In two-step marking procedures, a clip is placed before NACT, and a different, intra-operatively identifiable marker is placed before surgery. Difficulties may arise when visualizing the clipped lymph node for secondary marking.

With two-step marking procedures, Balija et al. reported IR to be 79.2% in 77 patients. Here, a clip was placed before NACT, and a SAVI SCOUT marker or a hook-wire was placed within the last 8 weeks before surgery.^31^ In a similar study reporting on clip marking before NACT and SAVI SCOUT before surgery, the SAVI SCOUT marker and the clip were found in different lymph nodes in 13% of the patients.^34^ Another study examined two-step marking with a clip placed at the beginning of NACT and a ^125^I seed marker placed in the MLN 5 days before surgery. Here, identification of the ^125^I MLN upon surgery was successful in 34/35 patients. However, the clip could not be ultrasonographically visualized in two patients at marking before surgery, and the radiologist had to rely on visual characteristics of the lymph node.^21^

The challenges with two-step marking procedures are partly contradicted in a study by Simons et al. Here, one group had a ^125^I seed placed before NACT and another group had a clip and a ^125^I seed or hook-wire placed after NACT. In this series, the reported overall IR of the MLN was 92.8%, and no difference in IR between groups were found.^35^ In the abovementioned study by Martinez et al., they also compared the group with Magseed® marking before NACT with a group consisting of 37 patients who had a coil placed before NACT and Magseed® placed after NACT. They found no difference in IR or FNR between the two groups.^32^

In our study, 41% of the patients achieved ax-pC. Rates vary with the receptor profile of the primary tumor, with the highest ax-pCR in patients with ER^-^/ HER2/*neu*^+^ tumors. Other studies have shown ax-pCR ranging from 31 to 63%.^1–8,33^ These high rates of ax-pCR underline the importance of de-escalating axillary surgery in these patients, as patients with ax-pCR are not expected to benefit from ALND.^36^

We found the MLN to be an SN in 72% of the patients. Other studies have shown that the MLN is an SN in 61–96% of the patients.^1,6,16,19,32,34,37^ Variability in concordance rates may be explained by the fact that accessibility of the lymph node plays a role in deciding which lymph node to biopsy and mark in case of more than one suspicious-looking lymph node. This may increase the likelihood that the MLN is not an SN. Patients where the MLN is not an SN risk inferior axillary staging if staged with SLNB or excision of an MLN alone.

Here, if patients were staged with SLNB only, 79.5% of patients with metastases in the lymph node would have been detected. Excision of an MLN only would yield a 94% detection rate of residual metastatic disease. This points towards an improved detection rate of residual disease when combining SLNB and the excision of an MLN. Excision of at least three SNs as an axillary staging method, as recommended by others, would have been possible in only 26% of the patients in this study, lower than reported elsewhere.^9^ In 12 patients, no SNs were found. This yields an SN IR of 91.5%, which is in accordance with the RISAS study mentioned above.^30^

This project was designed to evaluate the feasibility of ^125^I marking. Limitations include the partly retrospective design. The results are based on a multicenter cohort consisting of all Danish patients treated with TAD with long-term ^125^I marking since the introduction of the procedure in 2016. Danish guidelines discourage ALND in the case of ax-pCR, and therefore we cannot assess the FNR in this cohort. Contributing urgently needed oncological safety data, the ongoing MINIMAX study (clinicaltrials.gov ID NCT04486495) has disease-free and overall survival after less invasive axillary staging techniques as primary endpoints.^38^ However, results are not expected before 2023. The AXSANA study (clinicaltrials.gov ID NCT04373655) run by EUBREAST, where different surgical methods of axillary staging in clinically node-positive breast cancer patients treated with neoadjuvant chemotherapy are investigated, is also expected to contribute knowledge to this field.^13^ Simultaneously, the MAGELLAN trial (clinicaltrials.gov ID NCT0396559) investigates the feasibility of one-step marking with MagSeed®.

One point in one-step ^125^I seed marking is that it is not yet known whether the gamma radiation emitted by the ^125^I seeds may influence small lymph node metastases by itself, leading to pCR in the ^125^I marked node alone. Due to this, concerns could be raised if FNR is higher after ^125^I marking than other marking methods, which may need further investigation. Additionally, as a radioactive source, radiation hygiene measures concerning these seeds need consideration, e.g., when placing seeds at the time of biopsy without a verified positive lymph node, as these seeds need removal regardless of biopsy histology.

In summary, one-step ^125^I seed marking is highly reliable when considering IR after NACT and provides an easy marking method with no need for re-marking before surgery. Since rates of ax-pCR vary widely depending on tumor receptor profile and a large proportion of patients have different SN and MLN, staging with TAD should be considered as an option for certainty about nodal status after NACT. Survival studies would add to the knowledge about axillary staging in the NACT setting.
